# The Book World of Medicine and Science

**Published:** 1902-06-28

**Authors:** 


### THE BOOK WORLD OF MEDICINE AND SCIENCE.

A HERO OF DONEGAL: a Memoir of Dr. William Smyth of Burtonport. By FREDERICK DOUGLAS HOW. With Eight Illustrations. (London: Isbister and Co., Limited. Price 2s. 6d. net)

THE public memory is so short, that it is likely that many people have already forgotten Dr. William Smyth, of Burtonport, in Donegal, who died of typhus fever, which he had contracted while tending, nursing, and removing, practically single-handed, patients suffering from that disease in the island of Arranmore. This brief and most interesting memoir will recall the name of one of those heroes for whom life offers no reward, and yet of whom this empire has never had any lack. In the autumn of 1901 he was told of some cases of typhus fever in the island of Arranmore, which was within his district. So great was the fear of infection that neighbours would do nothing for the sick family, and the doctor had to attend to all the details of nursing, and bring the food and comforts his patients required from his own home. He managed to get the patients to consent to go to hospital, but had great difficulty in managing their removal. The hospital authorities were willing to send the ambulance to the nearest point on the mainland, but between that and the fever-stricken hut lay three miles of sea. The doctor's own yacht was too large for him to manage alone, and his other boat, a punt, was too small to convey the four patients who were to be removed. Of the fishermen who were his most devoted helpers in most things, not one would lend a boat for such passengers, and he had to apply to the Local Board for permission to purchase an old boat in which to remove the patients, and was allowed to buy an unseaworthy craft which had not been in the water for two years. Aided by another brave man, Dr. McCarthy, of Londonderry, Medical Officer of Health for Donegal, Dr. Smyth conveyed the sick people to the ambulance; but he himself had by this time the germs of the disease in his system, and in a few days he took to his bed, never to rise again. A fortnight later he died, at the age of 42, leaving a wife and eight children who were, as was not strange under the circumstances, but ill-provided for. Such is the record of a brave man's life. Besides the strictly biographical part of it, Mr. How's book contains many interesting touches illustrative of the people among whom William Smyth spent his life. One "wake" story is so good that we quote it. Bridget O'Brenan died. She had long been bent almost double with age and infirmity, and in order to straighten her out for her coffin heavy stones were laid upon the corpse. While the wake was proceeding, one of the company, waving his whisky-glass, shouted out, "Here's a health to you, Bridget O'Brenan." As he spoke Bridget jumped up, and in a moment the house was empty of all but herself. Not that the poor old dame had returned to life, as did Conn in "The Shaugraun," but the stones had slipped off the body, and their weight removed, the body had suddenly resumed its wonted attitude. This book deserves to be widely read, and will repay the reading.

TUBERCULOSIS AS A DISEASE OF THE MASSES AND HOW TO COMBAT IT. Prize Essay by S. A KNOPF, M.D., adapted for use in England by J. M. BARBOUR, M.B. (London: Rebman, Limited. 1902. Price 1s. net.)

AT a meeting of an international congress held in Berlin in 1899 a prize of 4,000 marks was offered by two Berlin merchants for the best essay on the subject "Tuberculosis as a Disease of the Masses and how to Combat it," and this is the essay that gained the prize. Of course it is of a popular character, but it is very well done, as anyone would expect to be the case who knows the position occupied by Dr. Knopf in relation to the question dealt with. The whole treatment of the subject may be regarded as a logical application of the acceptance of the infective nature of tuberculosis. After stating broadly the bacillary nature of consumption, the author describes the various methods by which attempts may be made to prevent the dissemination of the infective germs. Coming to the question, What protects healthy individuals from the infection? the whole problem of hereditary and acquired predisposition is carefully gone into; occupations, forms of dress, rearing of children, and modes of life are discussed; then a full description is given of the sanatorium treatment of the disease; and lastly the political and sociological aspects of the matter are discussed. It is pointed out that in addition to what might be done to remove conditions leading to tuberculosis, the tide of emigration from village to city should be reversed; statesmen should protect the interests of the farmer, so that farming will have more attraction to the rising generation than it has had in the last few decades; and philanthropists should aid the statesmen by endowing institutions for instruction in scientific and profitable agriculture, and also in providing healthful amusements, good libraries, and other educational institutions in country places, so as to make life outside large cities more interesting and attractive to young people. The tuberculosis question evidently touches some of the problems in sociology which are at the same time the most interesting and the most disputed. This is an admirable essay.

THE HUMAN BODY, ITS PERSONAL HYGIENE AND PRACTICAL PHYSIOLOGY. By BUEL P. COLTON. (London: Scientific Press. Price 5s.)

THIS is a sort of superior school book for advanced classes, dealing primarily with human physiology and, taking that as a basis, with hygiene. This is the proper order in which to study these subjects—first the laws of life then the laws of health. It has been far too common a practice to regard hygiene as a branch of medicine, a thing to be studied professionally by doctors, and by the general public to be left alone. Here we find it in its proper place as a practical application of the teachings of physiology. Contrary to what is found in many popular handbooks, which begin by a description of the skeleton, gradually clothe the bones with flesh, and finally insert the internal organs, the author of this book begins with protoplasm and then going on to nerves and muscles and functions. He thus works through the general principles of physiology, giving enough anatomy as he goes on to make the descriptions clear, and only finally in the last chapter gives a description of the human skeleton as a whole. There is no doubt that this is by far the most interesting and instructive method of dealing with the subject. Beginning with the organs of locomotion, we find a good description of muscular action and of the relations of the muscles to the moving parts; then we come to the nervous system and its relation to motion, sensation, and reflex action, the whole being described in accordance with the older hypotheses and without reference to neurons. Then to blood and its circulation; to respiration and the changes it involves; to excretion and digestion; to foods and foodstuffs, and all the phenomena connected with assimilation and nutrition. After all this we come back to the nervous system as displayed in the brain and the special senses. Interspersed throughout the various chapters there is a good deal of information on many outside but cognate matters related to hygiene, while there is a special chapter dealing with what one may call first aid and nursing emergencies, together with useful appendices on foods and cooking, antiseptics, and statistical matters. The book is well and clearly written, and is admirably produced, the illustrations (coloured and other) being both numerous and excellent.

